# The Pattern of Variation between Diarrhea and Malaria Coexistence with Corresponding Risk Factors in, Chikhwawa, Malawi: A Bivariate Multilevel Analysis

**DOI:** 10.3390/ijerph120708526

**Published:** 2015-07-21

**Authors:** Salule Masangwi, Neil Ferguson, Anthony Grimason, Tracy Morse, Lawrence Kazembe

**Affiliations:** 1 Centre for Water, Sanitation, Health and Appropriate Technology Development (WASHTED), University of Malawi, The Polytechnic, P/B 303, Blantyre, Malawi; E-Mail: tracythomson@africa-online.net; 2 Department of Mathematics and Statistics, University of Malawi, The Polytechnic, P/B 303, Blantyre, Malawi; 3 Department of Civil Engineering, University of Strathclyde, Glasgow G4 0NG, UK; E-Mail: n.s.ferguson@strath.ac.uk; 4 Scotland Chikhwawa Health Initiative (SCHI), P.O. Box 30376, Blantyre 3, Malawi; E-Mail: a.m.grimason@strath.ac.uk; 5 Africa Academy for Environmental Health, P.O. Box 15574, Sinoville 0129, South Africa; 6 Department of Statistics and Population Studies, 340 Mandume Ndemufayo Avenue, Pionerspark, P/Bag 13301, Windhoek, Namibia; E-Mail: lkazembe@yahoo.com

**Keywords:** malaria and diarrhea coexistence, random effects, Bayesian Analysis, bivariate multilevel analysis, household and community variation, Southern Malawi

## Abstract

Developing countries face a huge burden of infectious diseases, a number of which co-exist. This paper estimates the pattern and variation of malaria and diarrhea coexistence in Chikhwawa, a district in Southern Malawi using bivariate multilevel modelling with Bayesian estimation. A probit link was employed to examine hierarchically built data from a survey of individuals (n = 6,727) nested within households (n = 1,380) nested within communities (n = 33). Results show significant malaria [$\sigma_{u1}^{2}=0.901$ (95% CI:0.746,1.056)] and diarrhea [$\sigma_{u2}^{2}=1.009$ (95% CI:0.860,1.158)] variations with a strong correlation between them [${\text{r}}_{\text{u}}^{\left(1,2\right)}=0.565$] at household level. There are significant malaria [$\sigma_{v1}^{2}=0.053(95\%\;CI:0.018,0.088)$] and diarrhea [$\sigma_{v2}^{2}=0.099(95\%\;CI:0.030,0.168)$] variations at community level but with a small correlation [$r_{v}^{\left(1,2\right)}=0.124$] between them. There is also significant correlation between malaria and diarrhea at individual level [${\text{r}}_{\text{e}}^{\left(1,2\right)}=$0.241]. These results suggest a close association between reported malaria-like illness and diarrheal illness especially at household and individual levels in Southern Malawi.

## 1. Introduction

While the World Health Organisation Millennium Development Goals (MDGs) propose to reduce the burden of morbidity and mortality by 2015 [1,2], establishing the cause of disease or death may not be straightforward. The complex nature of underlying risk factors, often leading to concurrent infections in sick individuals [3], is a challenge in many settings. In developing countries, this phenomenon is highly prevalent at the community level and among children seeking care [4]. The problem of concurrent infection occurs due to overlapping risk factors such as nutrition, HIV/AIDS, and overcrowding [5]. Malaria and diarrhea are some of the diseases that share common risk factors in tropical developing regions. Deaths can be prevented if interventions are targeted at either one or a pair of concurrent diseases [6,7]. Traditionally programmes for child care mainly addressed single diseases such as malaria, diarrhea, and acute respiratory infections [4]. 

As Fenn *et al.* [6] observed, concurrent diseases in communities should be matched with combined prevention strategies of the diseases and that if the diseases act synergistically families must be properly advised and equipped to combat the problem. The Integrated Management of Childhood Illness (IMCI) programme was established in the mid 1990s by the World Health Organisation (WHO) and United Nations Children’s Fund (UNICEF) with the aim, among others, of addressing such coexistence of diseases in children [3,4]. 

For programmes such as the IMCI to be successful empirical methods to determine characteristics of diseases that coexist are necessary. Joint analysis of such diseases has recently become more popular in identifying similar patterns of variation to provide evidence of clustering [5,8,9,10,11]. It has been observed that multivariate Bayesian models are more precise in dealing with many realistic epidemiological problems in comparison with those obtained with classical indirect standardisation [12]. This study employs a bivariate multilevel modelling technique with the aim of analysing the coexistence between malaria and diarrhea in Chikhwawa by identifying patterns of variation and their common risk factors.

Chikhwawa is a district in southern Malawi. It has a surface area of 4,755 Km^2^ and an elevation of only 100 m above sea level. Chikhwawa is faced with a number of environmental and socioeconomic problems that are responsible for infectious diseases. Malaria and diarrhea are amongst the most common causes of illness and death in the district [13]. Estimated morbidity due to malaria and diarrhea are at 53% and 24.4% respectively [14]. This is statistically higher than the national averages 41.7% for malaria and 18% for diarrhea [7,15].

## 2. Methods

### 2.1. Sample

Data was obtained from Chikhwawa from a representative sample of individuals, households, and villages. A two-stage survey methodology similar to that used in national surveys [16] was adopted to produce a representative sample of households and individuals in the district. The first-stage involved sampling of villages that were strategically selected with a probability proportional to the number of enumeration areas in each Traditional authority (Chikhwawa has eleven traditional authorities and each traditional authority has several villages under its jurisdiction). The second sampling stage took place on the day of interviews. Households were systematically chosen with equal probability sampling. 

Only the matriarchal figure (only most senior matrichal figures in the households were interviewed) from each household was interviewed. If a household had no matriarchal figure the enumerator proceeded to the next household with a matriarchal figure. After discarding missing and incomplete data a total of 6727 individuals nested within 1380 households from 33 communities were used in this study.

The survey was carried out in the month of September in 2007. Information was sought for the months of January 2007 through to September 2007. 

### 2.2. Measures

Each household member’s reported malaria-like and diarrhea illnesses were used as the outcome variables. Respondents were asked to report the number of times each member of their household had experienced an episode of either malaria episodes or diarrhea illness since January. If a member experienced at least an episode of either malaria or diarrheal illness a score of 1 was recorded otherwise a 0. Thus we obtained two dichotomised responses, one for malaria episodes and the other for diarrheal illness.

This study used the Malawi Ministry of Health guidelines to health workers that fever without another identifiable cause should be treated as malaria if accompanied by one of the following symptoms: headache, chills, shivering, or loss of appetite [17]. Thus any information on additional symptom of malaria to fever as indicated above or information of a test at a health facility, or if anti-malarial drugs cured an ailment was desirable to confirm a malaria episode. To reflect this description this study uses the term ‘malaria-like’ instead of ‘malaria’ in its analysis and discussion of the results. 

Also considering that diarrhea is often defined differently in various studies and countries [18], a standard definition of a diarrhea-day was used in this study, *i.e.*, one where a subject experiences three or more loose or watery stools in 24 hours or any number of loose or watery bloody stools [19,20]. In Malawian vernacular language diarrhea is known as *‘kutsegula m’mimba’* literally meaning opening up of bowels which is associated with defecation of watery stools especially in young children. However ‘bloody stools’ or dysentery, are known as *‘kamwazi’* assumed to be a different disease from diarrhea by other people. Interviewers were, therefore, advised to use both vernacular definitions of diarrhea and dysentery when collecting data on diarrheal illness.

**Table 1:** Descriptive estimates of risk factors of malaria and diarrhea in Chikhwawa, Southern Malawi, 2007.

Discrete variables					
Risk factor	Description	value	Number at risk	% infected	
				malaria	diarrhea
*Individual level*					
Age	0–5 yrs	1	1444	62.3	35.7
	6–10 yrs	2	1102	50.3	18.8
	11–20 yrs	3	1344	42.4	17.1
	21–40 yrs	4	2027	55.1	26.9
	41–60 yrs	5	573	59.9	29.0
	> 60 yrs	6	237	62.0	26.6
School	No school	1	2552	58.2	31.4
	Primary school	2	3576	50.6	22.9
	≥ Secondary	3	599	53.6	17.9
Sex	Male	1	3318	51.9	24.5
	female	2	3409	55.5	26.8
Expectant Woman	Yes	1	144	68.8	35.4
	No	0	6583	54.4	25.5
*Household level*					
OHH	No job	0	429	59.4	34.5
	Has a job	1	6298	53.4	25.1
Distance to river	1 km or less	1	2463	-	22.8
	1 km to 2 km	2	2871	-	25.8
	> 2 km	3	1393	-	30.4
Drinking water source	Public tap	1	1041	-	28.2
	Private tap	2	419	-	21.0
	OSDWS	3	4593	-	24.5
	UDWS	4	674	-	32.3
**Continuous variables**					
**Risk factor**	**Description**	**Mean**	**Std. dev.**	**Median**	**Range**
*Household level*					
Maternal age (years)	-	35	13	31	74
Household size	-	6	2	5	12
*Community level*					
CDE	-	0.46	0.16	0.46	0.62
CME	-	1.10	0.24	1.05	1.02

OHH—Occupation of head of household; OSDWS—Other safe drinking water sources; UDWS—Unsafe drinking water sources; Relative wealth index; CDE—Community diarrhea endemicity; CME—Community malaria endemicity.

Table 1 lists the risk factors that were included in the model. The risk factors were obtained by first checking the simple relation between each potential risk factor and the outcome variable of interest while ignoring all other variables. Only risk factors that were significant at *p* ≤ 0.2 with a Deviance Information Criterion (DIC) [21] reduction of at least 7 were selected for the final model. Individual age, individual school level, expectant mothers, and gender were included as individual (level one) predictor variables. Matriarchal age, household size, head of household employment status, drinking water source, distance to the nearest river (notice that distance to the nearest river and nearest health facility were included as household variables because households from the same community could have different proximities to the same nearest river or would have different nearest rivers and they could report to different health facilities based on distances and socioeconomic preferences), nearest health facility, household mosquito-net ratio, distance to the nearest stagnant water body, and relative wealth status were included as household (level two) predictor variables. Community’s malaria and diarrhea endemicity were included as community (level three) predictor.

Maternal age, household size, and diarrhea or malaria endemicity were continuous variables.

Household wealth index was derived using the method of ‘variations’ [19] that assigns weights to indicator variables and uses the inverse of the proportion of number of households with an asset or service as the weight for the indicator. A categorical variable was derived by cutting the wealth index distribution into three distinct segments [20] 

Diarrheal and malaria-like (ML) endemicity were derived by first computing the total number of sampled diarrheal and ML episodes. The results in each community were divided by the total number of sampled individuals in that community. 

### 2.3. Analysis and Estimation

The bivariate regression model [21,22] was used to explain the joint probability of binary malaria and binary diarrhea outcomes for individuals.

Assume *y_ijkq_*is the response of ML disease (q=1) and diarrhea illness (q=2) for individual *i* (i=1,....,6727) in household *j* (j=1,.....1380) and community *k* (k=1,...,33) such that

$$y_{ijkq}=\{\begin{array}{c} \begin{array}{ccccc} 1 & if & disease & q & exist \end{array}\\ \begin{array}{cccc} 0 & no & disease & q \end{array} \end{array}$$

then the bivariate 3-level hierarchical model for presence of ML disease and diarrheal illness is given by

(2) $$\left(\begin{array}{c} probit(p_{ijk1})\\ probit(p_{ijk2}) \end{array}\right)=\left(\begin{array}{c} \alpha_{1}\\ \alpha_{2} \end{array}\right)+X_{ijkq}^{T}\left(\begin{array}{c} \beta_{1}\\ \beta_{2} \end{array}\right)+\left(\begin{array}{c} u_{jk1}\\ u_{jk2} \end{array}\right)+\left(\begin{array}{c} v_{k1}\\ v_{k2} \end{array}\right)$$

where $\alpha_{q}=(\alpha_{1},\alpha_{2})$is the intercept for disease *q*, in individual *i* found in household *j* residing in community *k*. The term $\beta ={\left(\beta_{1},\beta_{2}\right)}^{T}$is a vector of regression parameters corresponding to a set of covariates $X_{ijkq}$. The components $u_{jkq}$ and $v_{kq}$ are the unstructured heterogeneity variation terms at the household and community levels respectively. Since the outcomes may be dependent then their error terms may be correlated such that if $\text{cov}(e_{ijk1},e_{ijk2})=\sigma_{e}^{\left(1,2\right)}$, $\text{cov}(u_{jk1},u_{jk2})=\sigma_{u}^{\left(1,2\right)}$, an $\text{cov}(v_{k1},v_{k2})=\sigma_{v}^{\left(1,2\right)}$are the covariance at individual, household, and community levels respectively and $\text{var}(e_{ijkq})=\sigma_{eq}^{2}$, $\text{var}(u_{jkq})=\sigma_{uq}^{2}$, and $\text{var}(v_{kq})=\sigma_{vq}^{2}$ are their corresponding variances then $r_{e}^{\left(1,2\right)}=\frac{\sigma_{e}^{\left(1,2\right)}}{\sqrt{\sigma_{e1}^{2}\sigma_{e2}^{2}}}$, $r_{u}^{\left(1,2\right)}=\frac{\sigma_{u}^{\left(1,2\right)}}{\sqrt{\sigma_{u1}^{2}\sigma_{u2}^{2}}}$, and $r_{v}^{\left(1,2\right)}=\frac{\sigma_{v}^{\left(1,2\right)}}{\sqrt{\sigma_{v1}^{2}\sigma_{v2}^{2}}}$ are the correlations between ML disease and diarrheal illness at individual, household, and community levels respectively.

Estimation was performed using Bayesian procedures in MLwiN 2.10 software. Stability of all model parameters was monitored by observing the Raftery-Lewis diagnostics [23]. The maximum number of iterations performed was 50,000.

## 3. Results 

This study attempts to identify common risk factors between ML episodes of disease and diarrheal illness and their corresponding pattern of variation at household and community levels. Table 1 provides descriptive statistics for hypothesized risk factors at individual, household, and community levels along with prevalence of ML and diarrhea. Slightly more than half and a quarter of the people in the sample had at least an episode of ML and diarrhea respectively within the study period. The mean ratio for ML episodes per person per village during this period was 1.10 (range: 0.57–1.60) and of diarrhea was 0.46 (range: 0.16–0.78). 

**Figure 1:**
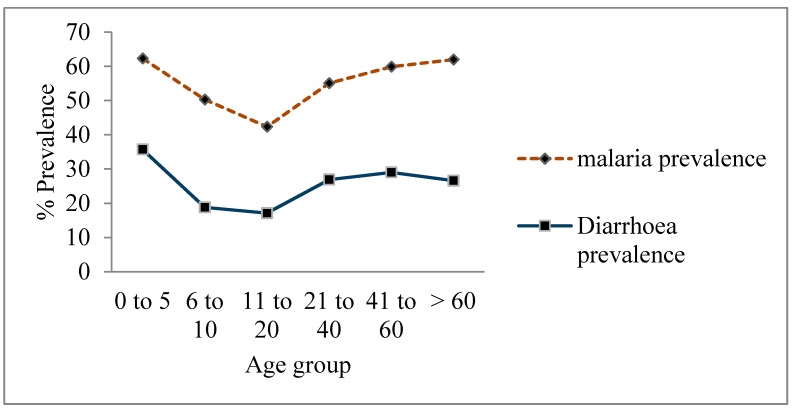
Curves showing the relationship between malaria and diarrheal illnesses with respect to age groups.

There is similarity in the pattern of risk factors between ML and diarrheal illnesses at all levels. Figure 1, for example, shows independent curves, one for ML and the other for diarrheal prevalence, depicting a similar relationship between the two diseases and individual age groups. There were significant Pearson Chi-square variations in age groups with respect to both ML and diarrheal illness ($\chi_{df=5}^{2}=124.4;p=0.000$; and $\chi_{df=5}^{2}=160.6;p=0.000$ respectively). There was also significant variation in individual school level with respect to both malaria-like ($\chi_{df=2}^{2}=34.6;p=0.000$) and diarrheal illness ($\chi_{df=2}^{2}=78.1;p=0.000$). The higher the level of school the less likely an individual was reported to have suffered from both diseases. Female individuals and expectant mothers had higher risks of suffering from both malaria (68.8%) and diarrhea (35.4%). 

At household level, families with low wealth index were more likely to get sick than those that had higher wealth indexes. Families whose head of household had no job were more at risk of contracting both ML and diarrheal illness. The median age of responsible mothers per household was 31 years (range: 15–89) and on average there were six people (range: 1–13) per household. 

### 3.1. Fixed Effects of Malaria and Diarrhea Morbidities

Table 2 provides Bayesian estimates of a bivariate 3-level hierarchical model between malaria-like and diarrheal illness. Individuals aged 6–40 were less likely to suffer from both malaria-like and diarrheal illness compared to infants and young children aged 0–5 years. Those aged above 40 years were more likely to suffer from ML symptoms when compared to all other age groups. There was no difference in diarrheal illness between children under the age of five and those older than 40 years. 

Expectant mothers were more likely to suffer from ML symptoms (β=0.437; 95% CI:0.143,0.731) and were marginally more likely to suffer from diarrhea than all other individuals (β=0.212;95% CI:−0.068,0.492;p=0.14). Females were more likely to suffer from ML than their male counterparts (β=0.103;95% CI:0.027,0.179). There was no difference in diarrheal illness between females and males. 

Members from families with older mothers were less likely to suffer from both ML (β=−0.011;95% CI:−0.017,−0.005) and diarrheal disease (β=−0.010;95% CI:−0.016,−0.004). There was a negative linear relationship between malaria and family size indicating that members from bigger families were less likely to suffer from ML illness than smaller families (β=−0.052;95% CI:−0.087,−0.017). There was a quadratic relationship between diarrheal disease and family size with a turning point at family size of six (Table 2).

Employment status of a head of a family was a significant risk factor for diarrheal illness but not ML disease. Families that had an employed head of household were less likely to suffer from diarrhea than those whose head of household was jobless [β=−0.380;95% CI:−0.668,−0.092].

Water source was a strong risk factor for diarrhea sickness. Households that used unsafe water sources (rivers, streams, and ponds) as drinking water in their homes were more likely to suffer from diarrhea than those that used private piped water [β=0.491;95% CI:0.056,0.926]. However, there was no difference in diarrheal prevalence between those that used public piped water, boreholes, springs, or protected wells and those that used private piped water.

At community level both ML and diarrheal endemicity were strong risk factors for both ML and diarrheal disease when placed in the model interchangeably. The higher the ML or diarrhea endemicity the more likely members of that community were likely to suffer from either diarrhea or ML disease. This observation shows that individuals from high malaria-like endemicity communities were likely to suffer from either ML or diarrhea illness and similarly for individuals from high diarrheal endemicity communities.

**Table 2:** Fixed effects estimates from the bivariate model of malaria and diarrhea coexistence in Chikhwawa 2007.

Fixed effects		Malaria		Diarrhea	
		β	95% CI	β	95% CI
Individual age	0 to 5 years	Reference category			
	6 to 10 years	−0.257	(−0.398, −0.116)	−0.576	(−0.733, −0.419)
	11 to 20 years	−0.498	(−0.647, −0.349)	−0.582	(−0.752, −0.411)
	21 to 40 years	−0.201	(−0.328, −0.074)	−0.243	(−0.382, −0.104)
	41 to 60 years	0.177	(0.001, 0.353)	0.001	(−0.187, 0.189)
	60 years above	0.474	(0.225, 0.723)	−0.038	(−0.314, 0.238)
School level	None	Reference category			
	Primary	−0.092	(−0.200, 0.016)	−0.142	(−0.260, −0.024)
	≥ Secondary	−0.021	(−0.193, 0.151)	−0.472	(−0.674, −0.270)
Sex	Male	Reference category			
	Female	0.103	(0.027, 0.179)	0.04	(−0.046, 0.126)
Expectant woman	No				
	Yes	0.437	(0.143, 0.731)	0.212	(-0.068, 0.492)
OHH	No job	Reference category			
	Has a job	−0.13	(−0.408, 0.148)	−0.380	(−0.668, −0.092)
Maternal age		−0.011	(−0.017, −0.005)	−0.010	(−0.016, −0.004)
Household size	X	−0.052	(−0.087, −0.017)	−0.294	(−0.417, −0.170)
	X^2^	-	-	0.022	(0.012, 0.032)
CDE		0.977	(0.328, 1.626)	1.737	(1.098, 2.376)
CME		1.369	(1.0613, 1.677)	0.551	(0.184, 0.917)
Distance to river	< 1 km	Reference category			
	1 to 2 km	-	-	0.013	(−0.021, 0.285)
	> 2 km	-	-	0.180	(−0.002, 0.362)
Drinking water source	Public piped water	Reference category			
	Private piped water	-	-	0.178	(−0.149, 0.505)
	Other safe water sources	-	-	0.257	(−0.104, 0.618)
	Unsafe water sources	-	-	0.491	(0.056, 0.926)

CI-Confidence interval.

### 3.2. Random Effects of ML and Diarrhea Morbidities

The degree of group level effects is given in Table 3. Community variation was estimated as $\sigma_{v1}^{2}=0.053(95\%\;CI:0.018,0.088)$ for ML illness and $\sigma_{v2}^{2}=0.099(95\%\;CI:0.030,0.168)$for diarrhea. For household variation, we estimated $\sigma_{u1}^{2}=0.901(95\%\;CI:0.746,1.056)$for ML and $\sigma_{u2}^{2}=1.009(95\%\;CI:0.860,1.158)$ for diarrheal disease. This demonstrates that there are significant differences in both ML and diarrheal prevalence at household as well as at community levels.

**Table 3:** Covariance structure from the bivariate model of malaria and diarrhea coexistence in Chikhwawa 2007^§^.

Hierarchy	Malaria (95% CI)	Diarrhea (95% CI)
*Individual level*		
Malaria	$\sigma_{e1}^{2}=1$	$r_{e}^{\left(1,2\right)}=0.241$
Diarrhea	$\sigma_{e}^{\left(1,2\right)}=0.241;(0.180,0.302)$	$\sigma_{e2}^{2}=1$
*Household level*		
Malaria	$\sigma_{u1}^{2}=0.901;(0.746,1.056)$	$r_{u}^{\left(1,2\right)}=0.565$
Diarrhea	$\sigma_{u}^{\left(1,2\right)}=0.539;(0.441,0.637)$	$\sigma_{u2}^{2}=1.009;(0.860,1,158)$
*Community level*		
Malaria	$\sigma_{v1}^{2}=0.053;(0.018,0.088)$	$r_{v}^{\left(1,2\right)}=0.124$
Diarrhea	$\sigma_{v}^{\left(1,2\right)}=0.009;(-0.026,0.044)$	$\sigma_{v2}^{2}=0.099;(0.030,0.168)$

^§^ 95% confidence intervals in parentheses and correlation coefficients in the upper triangle of each level.

The covariance and correlation associated with the variations between ML and diarrheal diseases at all levels are also presented in Table 3. At community level the covariance between ML and diarrheal illness is $\sigma_{v}^{\left(1,2\right)}=0.009(95\%\;CI:-0.026,0.044)$ with a correlation of $r_{v}^{\left(1,2\right)}=0.124$; at household level the covariance is $\sigma_{u}^{\left(1,2\right)}=0.539(95\%\;CI:0.439,0.639)$ with a correlation of ${\text{r}}_{\text{u}}^{\left(1,2\right)}=0.565$; and at individual level the covariance is $\sigma_{e}^{\left(1,2\right)}=0.241(95\%\;CI:0.180,0.302)$ with a correlation of ${\text{r}}_{\text{e}}^{\left(1,2\right)}=$0.241.

**Figure 2:**
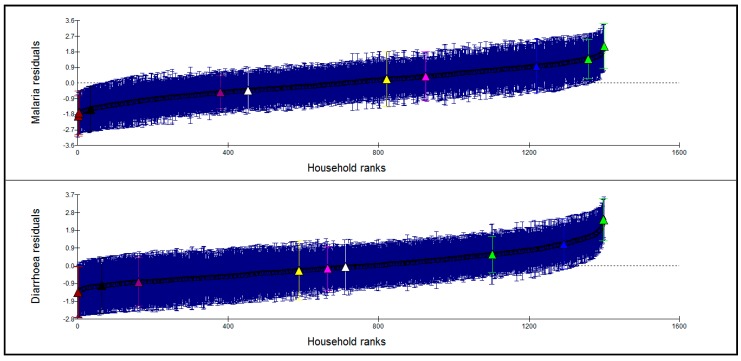
Caterpillar plot of household residuals for malaria and diarrhea prevalence. The dotted line is the mean of the estimated (shrunken) residuals, which is equal to zero (estimated or shrunken residual for group j is the residual obtained by multiplying the mean of the residuals of subjects in group j by a shrinkage factor. Shrinkage factor shrinks an observed group mean towards the centre of the population mean). The brushes represent 95% CI to the estimated residuals.

**Figure 3:**
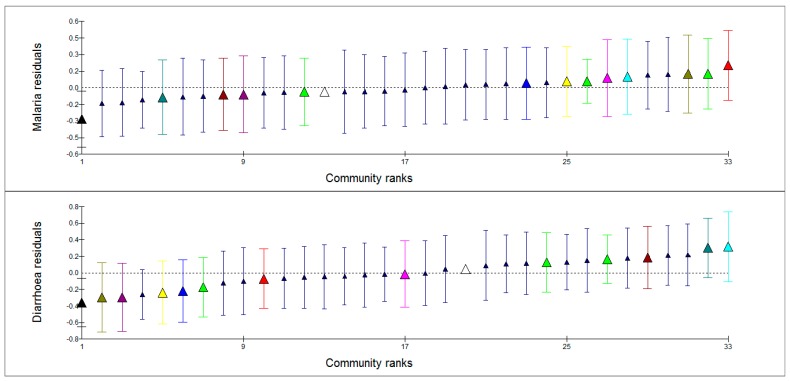
Caterpillar plot of community residuals for malaria and diarrhea prevalence. The triangles indicate estimated (shrunken) community residuals.

Figure 2 and Figure 3 show caterpillar plots of ML and diarrheal illness residuals at household and community levels respectively. A random sample of households (lower, middle and upper sections of the graphs) as highlighted on the graphs shows similarities of the pattern of variation of ML and diarrheal illness. Households with high ML prevalence were also with high diarrhea prevalence and vice versa. However, highlighted communities in Figure 3 show no clear pattern of variation with regard to ML and diarrheal prevalence. This demonstrates that there is a strong association between ML symptoms and diarrheal disease at household level and not at community level. There is also a significant relationship between the two diseases at individual level as indicated by the correlation factor. 

## 4. Discussion and Conclusions

In this study the aim was to determine the concurrence of reported ML disease and diarrheal illness in Chikhwawa, Southern Malawi by identifying patterns of variation and their common risk factors. This data accrued has shown significant variation both at community and household levels suggesting clustering of these two diseases in communities and families. The study also demonstrated significant correlation between reported ML disease and diarrheal illness within households (${\text{r}}_{\text{u}}^{\left(1,2\right)}=0.565$) and in individuals ($r_{e}^{\left(1,2\right)}=0.241$), indicating that households with more ML episodes also tended to have more diarrheal illness. Similarly individuals who experienced ML disease were also likely to have suffered from diarrheal illness during the same period. The analysis further suggests association between community ML disease or diarrheal illness with individual ML symptoms or individual diarrheal illness. This implies that individuals in communities with more ML disease were likely to have suffered from diarrheal illness; likewise individuals from communities with more diarrheal illness were likely to have suffered from ML symptoms. It follows that while ML endemicity in a particular community may influence individual ML or diarrheal illness outcomes in that community it does not necessarily affect other communities’ outcomes. These observations are similar to other studies that have observed similarities in the variation in childhood fever, diarrhea, and pneumonia due to shared and overlapping risk factors that include malaria endemicity [24]. 

After controlling for unique risk factors to malaria and diarrhea such as mosquito net ownership and drinking water sources respectively, other common risk factors between ML and diarrheal illnesses included age, school, sex, and pregnancy at individual level; maternal age, employment status of head of household, wealth status, and family size at household level; and either ML or diarrheal endemicity at community level.

The effect of age on ML disease and diarrheal illness is of particular interest and has been discussed in detail in another paper [25]. The increased likelihood of both malaria and diarrhea sickness in young children is attributed to underdeveloped immune systems in infants, poor breastfeeding practices, malnutrition, and lack of child health knowledge [26,27,28,29,30]. HIV/AIDS is prevalent in 14% of those between 15 and 49 years and waning of immunity in the elderly may explain the steady increase in risk of both malaria and diarrhea for those above 41 years old [30,31].

The low prevalence of ML and diarrheal disease amongst those that attended school may be due to the knowledge acquired from the school curriculum which addresses prevention and control of diarrhea and malaria [25,29] and provides increased awareness of existing healthcare resources [32,33,34]. The increased risk among female individuals may be due to induced stress brought about as a result of the heavy burden of household responsibilities placed upon women and young girls. The increased risk may also be due to greater exposure to mosquito’s and gastro-enteric pathogens as they go about fulfilling daily household chores e.g. utilising stagnant and/or contaminated water sources for chores such as dish-washing, laundry, and bathing [35,36]. 

Another significant risk factor to both ML disease and diarrheal illness in this study is pregnancy. The relationship between malaria and pregnancy has been a focus of research in Africa and it is a priority area in Roll Back Malaria strategy [37]. Pregnant women constitute the main adult risk group for malaria such that 80% of deaths due to malaria in Africa occur in pregnant women and children below five years. Immunity is normally low in pregnant women and their risk to infection is greater compared with women who are not pregnant living in the same area [38].

The relationship between matriarchal age and household ML or diarrheal illness is such that the risk of illness declines with increasing matriarchal age. This tendency has been linked to the experience and knowledge gained by matriarchal figures over many years of family caring and dealing with both malaria and diarrheal diseases [25]. Another explanation links elderly mothers to families with adults only who are less likely to have suffered from the two diseases compared with households with young mothers and children. 

Household size has a positive quadratic relationship with diarrheal prevalence and a negative linear relationship with ML prevalence. The decreasing prevalence of diarrhea with increasing family size from one to six family members and the negative linear relationship between ML illness and family size may reflect increasing experience in family management as the family expands. However, the impact of overcrowding [39] in families with more than six members overcomes all other factors and increases the chance of diarrheal illness. 

There is a limitation to this study that relies on self-reporting by a single person of information for all household members. The reporting may, as a result, be highly correlated with the mother’s literacy levels, ability to recall events, and perceptions on health status. Maternal variables plus household clustering that have been included in this model would help overcome this shortfall. Eight months of retrospective reporting by mothers may result in biases due to incomplete responses, forgotten episodes, and unrepresentative individual data. As no data was available from households without a matriarchal figure this may create bias. During the survey the mothers were not given a precise definition of what constitutes an episode of malaria or diarrhea. Therefore, the questions relied on the mother’s perception other than clinical or actual definitions. This may create variations among different households and villages because perception of an illness episode is not the same across different groups of people. Further most mothers identified malaria from fever which may be a symptom for other diseases other than malaria. To reduce the effect of these methodological limitations, questionnaires from each enumerator were carefully audited after each day’s survey and data was screened to ensure consistency of approach to questioning and responses and to determine if the data conformed to expected patterns. Respondents were also required to explain in detail why they thought a member’s illness constituted malaria other than through fever. Any additional symptom of malaria to fever or a test at a health facility was desirable to confirm a malaria episode.

The survey required mothers to recall information of up to eight months from January to September 2007 with the aim of capturing data that included the peak of the rain season when malaria and diarrheal morbidity are at their highest. There was a risk that some episodes would not be reported due to the length of the recall period, particularly when the illness was not extreme. However, since the aim of the survey was mostly to understand factors that influence malaria and diarrheal morbidity in individuals, families and communities of Chikhwawa, this risk was overlooked on the basis that the information obtained would outweigh the discrepancies in forgotten episodes. Moreover, other studies have concluded that more easily observed symptoms are less likely to suffer from selective reporting [5,40]. Recall bias is reported to be related to level of mother’s education, with more educated mothers most likely to remember and distinguish symptoms for most illnesses, therefore controlling for mother’s education in the analysis may capture a large part of the self-selective nature of reporting [5,41]

In general the bivariate multilevel analysis has shown that there is a close association between reported malaria-like illness and diarrheal illness especially at household and individual levels in Southern Malawi. Significant malaria and diarrhea variations have been highlighted both at community and household levels. Significant correlation between malaria and diarrhea has also been observed both at household and individual levels. 
